# Residue Degradation and Risk Assessment of Difenoconazole and Its Metabolite during Tea Growing, Processing and Brewing by Ultra-Performance Liquid Chromatography–Tandem Mass Spectrometry Determination

**DOI:** 10.3390/foods13071123

**Published:** 2024-04-07

**Authors:** Min Wang, Yating Ning, Yue Hu, Xinyi Cui, Fengjian Luo, Li Zhou, Miao Yu, Xinzhong Zhang

**Affiliations:** 1 Research Center of Quality Safety for Agricultural Products, Tea Research Institute, Chinese Academy of Agricultural Sciences, Hangzhou 310008, China; wmheyjj@126.com (M.W.); ningyating@tricaas.com (Y.N.); huyue@tricaas.com (Y.H.); lfj@tricaas.com (F.L.); lizhou@tricaas.com (L.Z.); 2 College of Horticulture and Landscape, Tianjin Agricultural University, Tianjin 300384, China; xycui2005@126.com; 3 Zhejiang Provincial Plant Protection Quarantine and Pesticide Management Institute, Hangzhou 310020, China; 4 Graduate School of Chinese Academy of Agricultural Sciences, Beijing 100081, China; 5 Key Laboratory of Tea Quality and Safety Control, Ministry of Agriculture and Rural Affairs, Hangzhou 310008, China

**Keywords:** tea, difenoconazole, metabolite, degradation, processing, ultra-performance liquid chromatography–tandem mass spectrometry (UPLC–MS/MS)

## Abstract

Residue dissipation and risk assessment of difenoconazole and its metabolite difenoconazole-alcohol during tea growing, processing, and brewing was first investigated by ultra-performance liquid chromatography–tandem mass spectrometry (UPLC–MS/MS). The limits of quantification for both difenoconazole and difenoconazole-alcohol were 0.001 mg/kg in fresh tea leaves and tea, and 0.0002 mg/L in tea infusion. In field trials, the dissipation half-lives of difenoconazole in fresh tea leaves was 1.77 days. After spraying, the residues of difenoconazole-alcohol increased and then gradually dissipated like difenoconazole. After 14 days, the dissipation rates of difenoconazole and difenoconazole-alcohol reached 99%. When fresh tea leaves were harvested on different days, the total processing factors (PFs) of difenoconazole and difenoconazole-alcohol for green tea were 0.86–1.05 and 0.78–0.85, respectively, while the total PFs for black tea were 0.83–1.13 and 0.82–1.66, respectively. Metabolism of difenoconazole was accelerated during tea processing. When brewing black tea, the leaching rates (LRs) of difenoconazole and difenoconazole-alcohol were 8.4–17.9% and 31.8–38.9%, respectively, while when brewing green tea, the LRs were 15.4–23.5% and 30.4–50.6%, respectively. The LRs of difenoconazole and difenoconazole-alcohol in black tea were higher than those in green tea. The potential threat to human health for dietary intake of difenoconazole and difenoconazole-alcohol residues from tea consumption is negligible. However, the dietary risk of difenoconazole in fruits and vegetables that are essential for daily diets is concerning, with a risk probability of 158%.

## 1. Introduction

Tea (*Camellia sinensis* L.) is a non-alcoholic beverage with a unique flavor, and it is loved by people all over the world for its wonderful taste and wide variety, which makes it a very important economic value for many producing countries. Tea planting not only meets the demand of the tea market but also brings huge economic benefits, there are also many problems that need to be solved in the process of large-scale tea planting, such as tea pests and diseases on tea tree survival and tea quality. Chemical pesticide control is the main tool to solve this problem. Difenoconazole is a type of triazole fungicide that has many applications in the treatment of tea and other crop pests and diseases, because of its broad-spectrum and high-efficiency ability to control a variety of fungal diseases [1,2].

Difenoconazole has been popular since its launch, with more than 300 registrations since its registration in China in 1999. Although it is an efficient fungicide, at the same time, there is a certain risk of residues in plants and the environment [3,4,5], its metabolites are also toxic to the environment or organisms, and this series of problems has gradually attracted the attention of scholars [6]. Difenoconazole-alcohol is the main environmental metabolite of difenoconazole, and the structures can be seen in Supplementary Figure S1. These environmental and biological toxicities will enter the human body through diet and other means [7,8]. Thus, the residues of difenoconazole in food have become the focus of research: Gao investigated the dynamics and residues of difenoconazole applied on lettuce and pak choi, and a dietary risk assessment using the risk quotient (RQ) was conducted and figured out that the risk of difenoconazole at seven interval days in leafy vegetables was acceptable [9]. Soudamini Mohapatra determined the residue dynamics of difenoconazole on pomegranate, the degradation half-life of difenoconazole was 6.4–8.4 days and the residues were only on the skin of pomegranate, and no difenoconazole residues were detected in the edible part of pomegranate [10]. Kaushik Banerjee examined the residues of difenoconazole in grapes and the pre-harvest interval (PHI) values of difenoconazole were 25.5 and 38.5 days at the recommended application dose and double application dose, respectively, and the residues of difenoconazole in all samples were below the maximum residue limit (MRL) [11]. Zhang established an analytical method for the simultaneous determination of difenoconazole residues in wheat straw, wheat grain, and soil, and the half-lives of difenoconazole in wheat straw were 3.6–5.5 days and in soil were 4.9–5.8 days, while the residues of difenoconazole in wheat grain were less than 0.01 mg/kg [12]. The effects of different canned tomato manufacturing steps, including washing, peeling, homogenization, simmering, and sterilization, on the residues of difenoconazole were studied and determined by UPLC-MS/MS. The results revealed that the washing and peeling process had a 99% reduction in difenoconazole residues, while other stages had little effect [13]. In the above studies, the residues of difenoconazole in different plants and environment samples were measured, and proved that most of them were attached to the surface and the risk could be avoided to some extent by washing and peeling processes, etc.

However, as a special edible plant and the special consumption [14], it is a very important question whether tea leaves will cause harm to human beings from the pesticide residues, especially of difenoconazole. After studying the dietary risk assessment of difenoconazole residues in tea and tea infusion, the factors influencing the leaching rates (LRs) of difenoconazole in tea infusion were investigated by a gas chromatography–electron capture detector (GC-ECD). The results indicated a negative correlation between the residue levels of difenoconazole and the sampling interval extension after application, with the safe interval for tea picking at 5 days. The LRs of difenoconazole in tea infusion were related to the brewing water temperature and the brewing time, and it was limited and primarily remained in the spent tea [15,16]. Also, the technology of surface-enhanced Raman spectroscopy was used to explore the rapid and large-scale detection of pesticide residues of difenoconazole in tea [17]. The residue definition of difenoconazole by JMPR for compliance with maximum residue limit (MRL) and for dietary intake for plant commodities is difenoconazole itself. However, due to uncertainty in the metabolic behaviors of pesticides [18], some pesticides can produce more hazardous by-products or metabolites during processing and even raise the residue amounts due to their concentration and substrate [19]. Previous studies had only investigated the residues of difenoconazole in tea but did not involve the residues of metabolites for difenoconazole and elimination in tea. Therefore, further study on the residue degradation metabolism pattern, metabolites, and consumption risk of difenoconazole during tea processing is necessary.

In order to investigate the residual degradation of difenoconazole in tea, the residue determination method of difenoconazole in different tea and tea infusion matrices, combined with a BondElut C_18_ solid phase extraction (SPE) column to enrich and purify the tea infusion, has been established using UPLC-MS/MS in our research team [12]. So, in this study, this method was validated and used to determine the residual degradation patterns of difenoconazole and its metabolite during tea growth, processing, and brewing process.

## 2. Materials and Methods

### 2.1. Materials and Reagents

The difenoconazole and difenoconazole-alcohol standard were obtained from Shanghai ANPEL Laboratory Technologies Inc. (Shanghai, China). Difenoconazole suspension concentrate (SC, 40%) was provided by DuPont Trading (Shanghai) Co., Ltd. (Shanghai, China). Analytical-grade sodium chloride (NaCl) and magnesium sulfate (MgSO_4_) were provided by Shanghai Lingfeng Chemical Reagent Co., Ltd. (Shanghai, China). HPLC-grade methanol (MeOH), acetonitrile (ACN), and benzene were provided by Merck KGaA (Darmstadt, Germany). HPLC-grade ammonium acetate (AMA) was acquired from Shanghai ANPEL Laboratory Technologies Inc. (Shanghai, China). LC–MS-grade formic acid (FA, ≥99%) was acquired from Shanghai Macklin Biochemical Co., Ltd. (Shanghai, China). The BondElut C_18_-SPE column (500 mg/6 mL) and Cleanert TPT-SPE column (1 g/6 mL) were purchased from Agela Technologies Ltd. (Tianjin, China). All pure water (H_2_O) was purchased from Wahaha Group (Hangzhou, China).

### 2.2. Instruments and Conditions

A Waters UPLC-MS/MS system equipped with an ACQUITY UPLC H-Class binary solvent manager, an ACQUITY UPLC FTN sample manager, an ACQUITY UPLC column manager, and a Waters Xevo TQ-S Micro triple quadrupole mass spectrometer with an electrospray ionization (ESI) source (Waters Corp., Milford, MA, USA) was used for the residue determination of difenoconazole and difenoconazole-alcohol. System control, data collection, and data analysis were performed by MassLynx 4.1 software workstation.

An ACQUITY HSS T3 column (100 mm × 2.1 mm, 1.8 μm; Waters, USA) was used with the mobile phases consisting of MeOH with 0.1% FA (A) and 10 mmol/L AMA in H_2_O (B) at the gradient chromatographic program shown in Table 1. The flow rate, column temperature, and injection volume were at 0.2 mL/min, 40 °C, and 5 μL, respectively. Multiple reactions monitoring (MRM) and ESI positive mode were applied for the residue determination of difenoconazole and its metabolite difenoconazole-alcohol in samples, with the capillary voltage set at 3.5 kV, source temperature at 150 °C, and desolvation temperature at 350 °C. The quasi-molecular ion ([M + H]^+^) of each compound was used for the precursor ion with dwell time of 0.1 s. Argon (Ar, 99.995%) was used as the collision gas, and nitrogen (N_2_, 99.5%) as the cone gas and desolvation gas at 50 L/h and 700 L/h, respectively. The gradient program for chromatographic elution and other tandem mass spectrum conditions by UPLC-MS/MS are shown in Supplementary Table S1.

### 2.3. Field Experiments and Sampling

Field trials were carried out in July–August 2021 at Shengzhou, Zhejiang, China (29°35′ N, 120°49′ E) according to the Guideline for the Testing of Pesticide Residues in Crops [20]. Three replicates were included in each plot area of 186 m^2^. A blank plot was used as a control group and the other two plots were used for the dissipation and final residual trials, respectively. An amount of 40% of difenoconazole SC was used in the dissipation trials and final residual trials at a dosage of 75 mL/ha with water of 750 L/hectare. Approximately 500 g of fresh tea leaves were collected for the dissipation experiments at intervals of 2 h (equal to 0.083 days), 1, 2, 3, 5, 7, 10, and 14 days after spraying, respectively. Before being sprayed, a portion of fresh tea leaves was collected, crushed, and stored as a blank sample in a refrigerator at −20 °C until analysis. About 3 kg of fresh tea leaves were collected for the final residual trails at intervals of 1, 5, 7, and 10 days (pre-harvest interval time, PHI) after spraying. The following different processing stages are used to process fresh tea leaves into green and black tea:

Steps in the processing of green tea: fixed fresh tea leaves at 220 °C for 10 min, rolled the fixed tea leaves at 25 °C for 30 min, and then dried in an oven at 120 °C for 60 min. Steps in the processing of black tea: withered fresh tea leaves at room temperature for 6 h, rolled the withered tea leaves at 25 °C for 30 min, fermented at 35 °C for 6–8 h, and then dried at 120 °C for 60 min. Finally, the processed tea samples were ground and kept in a freezer at −20 °C for analysis.

### 2.4. Sample Preparation, Extraction, and Purification

First, weigh 5.00 g of fresh tea leaves (2.00 g of green or black tea) into a 50 mL centrifuge tube. Then, 20.0 mL of ACN was added (the dry tea samples were mixed with 10.0 mL of H_2_O with 1% of FA and placed for 30 min before adding ACN). The mixture was vortexed and sonicated for 10 min and placed for 30 min. After, 5.0 g of NaCl was added to the mixture and homogenized for 1 min by an IKA-T18 basic ultra-Turrax disperser. Then, the mixture was centrifuged for 5 min at 10,000 rpm, and 10.0 mL of supernatant solution was transferred into a heart-shaped bottle followed by evaporation at 40 °C to nearly dry, then redissolved by 5 mL of ACN/benzene (3:1, *v*/*v*) before being taken into the Cleanert TPT cartridge and eluted with 25 mL of ACN/benzene (3:1, *v*/*v*), then the cartridge was pre-conditioned with 5 mL of ACN/benzene (3:1, *v*/*v*). After that, the elution solvent was evaporated to dry at 40 °C and 1.0 mL of MeOH/H_2_O (1:1, *v*/*v*) was added to re-dissolve the residue, then the solution was filtered through a 0.22 μm filter film into an injection bottle for UPLC–MS/MS analysis.

A 3.0 g amount of tea was brewed in 150 mL of boiling water at a ratio of 1/50 to obtain the tea infusion according to the Methodology for Sensory Evaluation of Tea [21]. After pre-conditioning with 5 mL of MeOH and 5 mL of H_2_O, 100.0 mL of tea infusion (green tea or black tea infusion) was taken out for the BondElut C_18_-SPE cartridge cleanup. Then, 20 mL of MeOH was used to elute, and the elution solvent was collected and evaporated to dry at 40 °C. After resolving the residue with 1.0 mL of MeOH/H_2_O (1:1, *v*/*v*), the solution was filtered through a 0.22 μm filter film into an injection bottle for UPLC–MS/MS analysis.

### 2.5. Method Validation

The linearities, recoveries, limits of detection (LODs), limits of quantification (LOQs), and matrix effects (MEs) were verified. The linearities and correlation coefficients were calculated using the solvent standard solutions and matrix-matched standard solutions of difenoconazole and difenoconazole-alcohol with a concentration range from 0.001 to 5 mg/L. The calibration curves were plotted using the peak areas of the above substrates as the *y*-axis and their corresponding concentration levels as the *x*-axis. The formula [ME (in %) = (A/B − 1) × 100] is used for calculating ME, while A means the slope of the matrix standard curve (A) and B means the slope of the solvent standard curve (B). When ME ranges from −20% to 20% this means the matrix effect can be ignored, and if the ME is less than −20% this indicates a matrix inhibition effect, and if ME is greater than 20% this means a matrix enhancement effect. The LODs for both difenoconazole and difenoconazole-alcohol were determined using the lowest concentration with a signal-to-noise (*S*/*N*) ratio of 3.

The spiked recovery studies on fresh tea leaves, green tea, black tea, green tea infusion, and black tea infusion were used to evaluate the accuracy and precision of the residue analysis method of difenoconazole and difenoconazole-alcohol, respectively. To confirm the recoveries and relative standard deviations (RSDs), six replicates for each of four concentration levels spiked for fresh tea leaves, green tea, and black tea (0.001, 0.01, 0.10, and 1.00 mg/kg), and green tea infusion and black tea infusion (0.0002, 0.002, 0.02, and 0.2 mg/L) were set up. The lowest spiked concentration levels that met the requirements of recovery (70–120%) and relative standard deviation (less than 20%) were defined as the LOQs [22].

### 2.6. Data Analysis

The dissipation rates of difenoconazole and difenoconazole-alcohol on fresh tea leaves were used in the first-order kinetic equations to calculate the dissipation curves and half-lives according to Equations (1) and (2):

(1) $$C_{t}=C_{0}e^{-\mathrm{kt}}$$

(2) $$t_{1/2}=\ln2/k$$

In Equations (1) and (2), C_0_ is the initial residue (mg/kg) of difenoconazole or difenoconazole-alcohol on the fresh tea leaves. C_t_ is the residual amount (mg/kg) at interval t (day) after application. k is the dissipation rate constant. t_1/2_ is the half-life time (day) of difenoconazole or difenoconazole-alcohol.

The residue changes in difenoconazole or difenoconazole-alcohol in green tea (or black tea) before and after processing are expressed as the processing factor (PF) calculated by Equation (3):

(3) $$PF=R_{2}/R_{1}$$

In Equation (3), R_1_ and R_2_ indicate the residues of difenoconazole or difenoconazole-alcohol in green tea (or black tea) before and after processing, respectively. When the PF > 1 indicates an increase in residue in tea leaves after processing, and when the PF < 1 indicates that the residue in tea leaves was decreased after processing.

The residual transfer of difenoconazole or difenoconazole-alcohol at brewing is expressed as the leaching rate (LR) calculated by Equation (4):

(4) $$LR\;(\%)=R_{i}/R_{t}$$

In Equation (4), R_i_ indicates the residue (mg/kg) of difenoconazole or difenoconazole-alcohol in black tea infusion (or green tea infusion); R_t_ indicates the sum of residues (mg/kg) in the tea infusion from three brewing times and the spent tea leaves. The total LR is the sum of LRs for three tea infusions brewing.

Dietary intake risk assessment of difenoconazole or difenoconazole-alcohol in tea was conducted by the acute risks (RQa) and chronic risks (RQc); the calculation formulas are as follows:

(5) $$RQc=\frac{FI\;(kg)\times STMR\;(mg/kg)\times LR}{bw\;(kg)\times ADI\;(mg/kg)}\times 100\%$$

(6) $$RQa=\frac{FI\;(kg)\times HR\;(mg/kg)\times LR}{bw\;(kg)\times ARfD\;(mg/kg)}\times 100\%$$

In Equations (5) and (6), FI (kg) is the food intake amount; LR is the leaching rate; STMR is the median residue and HR is the highest residue in supervised trials; bw is the global average body weight (65 kg); ADI is the acceptable daily intake value, and ARfD is the acute reference dose, respectively. If the RQ value is higher than 100%, it means that the risk of difenoconazole or difenoconazole-alcohol to human exposure is unacceptable, whereas the RQ value is less than 100%, it means that the risk is acceptable [23].

## 3. Results and Discussion

### 3.1. Method Validation of Difenoconazole and Difenoconazole-Alcohol in Different Tea Samples

The linearities of the analytical method evaluated in the range of 0.001–5.0 mg/L for difenoconazole and its metabolite difenoconazole-alcohol in fresh tea leaves, black tea, and green tea, and black tea infusion and green tea infusion were shown in Table 1. The correlation coefficients (r^2^) of the standard curves for the different substrates were all greater than 0.9902. The MEs of difenoconazole and difenoconazole-alcohol in different matrices were from −74% to −51%, indicating the matrix suppression effects on various matrices and the matrix standard should be used for sample quantification. Additionally, in this case, the matrix suppression effect of difenoconazole was higher than difenoconazole-alcohol. The LODs in solvent, fresh tea leaves, green tea, and black tea matrices were 0.3 µg/kg, and in tea infusions were 0.07 µg/L, respectively. The UPLC–MS/MS chromatograms of difenoconazole and difenoconazole-alcohol in different matrices are shown in Supplementary Figure S2.

**Table 1:** Linear concentration ranges, regression equations, correlation coefficients (r), matrix effects (MEs), and limits of detection (LODs) for difenoconazole and difenoconazole-alcohol in different sample matrices by UPLC-MS/MS determination.

Compound	Sample Matrix	Concentration Range (mg/L)	Regression Equation	r	MEs %	LOD (µg/L)
Difenoconazole	ACN	0.0010~5.0	y = 2,489,640x + 10,259	0.9957	---	0.3
	Fresh tea leaves		y = 1,161,115x + 23,405	0.9990	−53	0.3
	Green tea		y = 699,291x + 20,874	0.9997	−72	0.3
	Black tea		y = 1,211,447x − 63,080	0.9913	−51	0.3
	Green tea infusion		y = 861,093x − 6849	0.9981	−65	0.07
	Black tea infusion		y = 954,850x + 68,794	0.9984	−62	0.07
Difenoconazole-alcohol	ACN		y = 1,197,384x − 10,872	0.9945	---	0.3
	Fresh tea leaves		y = 443,584x + 9557	0.9982	−63	0.3
	Green tea		y = 350,102x + 14,624	0.9995	−71	0.3
	Black tea		y = 481,152x − 29,396	0.9902	−60	0.3
	Green tea infusion		y = 348,690x + 6386	0.9991	−71	0.07
	Black tea infusion		y = 315,092x + 28,779	0.9968	−74	0.07

The average recoveries of difenoconazole and difenoconazole-alcohol at different spiked levels in all tea matrices were shown in Table 2, ranging from 73.4% to 118.6% with RSDs of 3.4–14.2%, and 71.2–114.9% with RSDs of 2.8–15.9%, respectively. The average recoveries of difenoconazole and its metabolite difenoconazole-alcohol spiked at the lowest spiked level were in the range of 70–120% with the RSDs below 20%, based on the Analytical Quality Control and Method Validation Procedures for Pesticide Residues Analysis in Food and Feed [22]. The LOQs for difenoconazole and difenoconazole-alcohol were 0.001 mg/kg in fresh tea leaves, green tea, and black tea, and 0.0002 mg/L in tea infusion, respectively. This proved that the method was accurate and feasible for the residue determination of difenoconazole and difenoconazole-alcohol in different tea matrices.

### 3.2. Residue Dissipation of Difenoconazole in Fresh Tea Leaves

**Figure 1:**
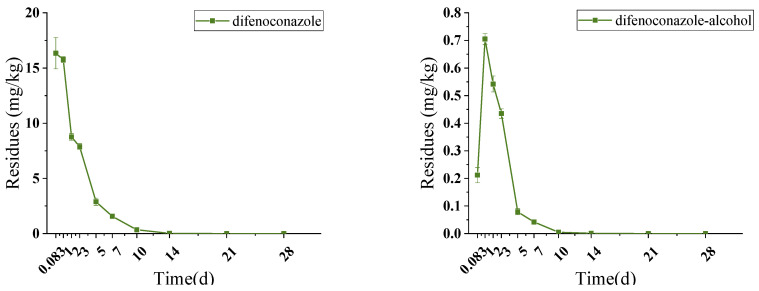
Dissipation trends of difenoconazole and difenoconazole-alcohol in fresh tea leaves.

Pesticides sprayed on tea plants not only leave residue but also have the potential to metabolize to low or even high toxic metabolites, which could lead to more serious safety problems although the residues degrade with time. The residue of difenoconazole in fresh tea leaves at 2 h (equal to 0.083 days in Figure 1) after spraying application was at 16.339 mg/kg, and then reduced to only 0.018 mg/kg in fresh tea leaves after 14 days with a degradation rate of 99.9%, shown in Figure 1 and Supplementary Table S2. Additionally, there was an increasing and then declining trend in the residue of metabolite difenoconazole-alcohol in fresh tea leaves, which increased from 0.212 mg/kg at 2 h to 0.705 mg/kg at 1 day, then gradually decreased to 0.001 mg/kg at 14 days with the degradation rate of 99.5%. At 14 days following spraying, the residues of difenoconazole and its metabolite in fresh tea leaves were significantly lower than the MRL of 0.05 mg/kg set by EU. Therefore, the safe pre-harvest picking interval of 14 days set for difenoconazole in tea gardens can effectively reduce the residue of difenoconazole in tea. The dissipation kinetic equation of difenoconazole in fresh tea leaves was C_t_ = 17.771e^−0.392t^ with R^2^ at 0.9641 and the half-life (t_1/2_) at 1.77 days. The dissipation rate of difenoconazole in fresh tea leaves is faster than that of other crops, as evidenced by the half-lives for difenoconazole at 10.1–11.4 days in celery leaves in Zhejiang and Shandong [24], at 6.6–7.8 days in cabbage [25]. This could pertain to the growth mechanism of fresh tea leaf growth [5], where pesticides applied in tea plants may be diluted due to the growth of tea buds, thus reducing the pesticide residues in tea [26].

### 3.3. Residue Transmission of Difenoconazole during Different Tea Processing

#### 3.3.1. Green Tea

**Figure 2:**
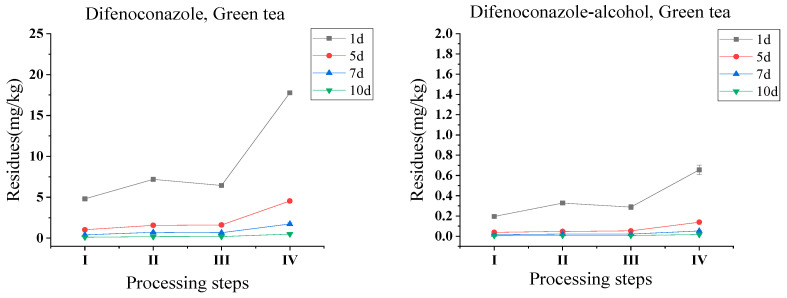

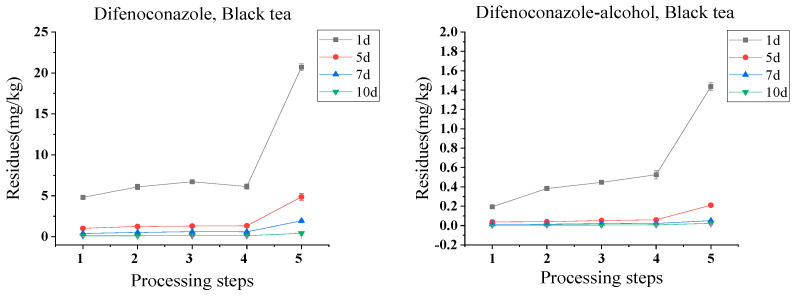
Residue variation in difenoconazole and difenoconazole-alcohol in various tea processing steps. Note: 1, 2, 3, 4, and 5 coordinates to the five steps of black tea processing, as fresh tea leaves, withering, rolling, fermentation, and drying, respectively; I, II, III, and IV coordinates to the four steps of green tea processing, as fresh tea leaves, fixing, rolling, and drying, respectively.

It is known that fresh tea leaves were not drunk directly, fresh tea leaves were often processed into different teas and then brewed for consumption, and the changes in pesticide residue and contents during the processing are more worthy of attention. Figure 2 and Supplementary Table S3 illustrate the residue variation in difenoconazole and its metabolite difenoconazole-alcohol in green tea after four processing steps of fresh leaves, fixing, rolling, and drying. The overall trend of difenoconazole residue in the samples was increasing during the processing steps, such as from 0.108–4.796 mg/kg to 0.180–7.186 mg/kg after fixing with processing factors at 1.50–1.75, after rolling dropped to 0.181–6.441 mg/kg with processing factors at 0.90–1.03, while after drying the residue rose to 0.483–17.776 mg/kg with processing factors at 2.64–2.82. The residual amount of difenoconazole rose more in the drying step because of the decrease in moisture content during drying. The trend of difenoconazole-alcohol during processing showed a similar upward trend to that of difenoconazole, rising slightly after fixing from 0.004–0.194 mg/kg to 0.007–0.327 mg/kg with processing factors at 1.24–1.75, then decreased slightly to 0.007–0.287 mg/kg after rolling with processing factors at 0.88–1.11, similarly to difenoconazole, the residues of difenoconazole-alcohol in the dried samples increased substantially to 0.016–0.655 mg/kg with processing factors at 2.28–2.65. Because the fixing and drying processes removed water from the fresh tea leaves, the residues of difenoconazole and difenoconazole-alcohol in tea samples increased during the processing of green tea.

Although each tea processing step contributes to the degradation and volatilization of pesticide residues, the loss of water in the sample during some processing thus leads to an increase in the level of pesticide residues [27]. Additionally, the drying process has a higher processing factor than other steps, probably because drying affects the water content of tea leaves most, which causes the pesticide residues to increase more [28]. Therefore, after conversion of the moisture content, the average degradation rate of difenoconazole from fresh tea leaves to dried tea was 4%, while the average elimination rate of difenoconazole-alcohol was 19%, the total processing factors of difenoconazole ranged from 0.86 to 1.05 (a.v.: 0.90), and difenoconazole-alcohol ranged from 0.74 to 0.87 (a.v.: 0.81). The most impact processing step on the variation in difenoconazole residues in green tea processing was fixing (PF = 0.89–1.11, a.v.: 0.99), followed by rolling (PF = 0.91–0.97, a.v.: 0.94), and drying (PF = 1.02–1.05, a.v.: 1.04), while the most influential processing step for difenoconazole-alcohol was fixing (PF = 0.70–1.04, a.v.: 0.92), followed by rolling (PF = 0.90–1.05, a.v.: 0.95), and the least influential was drying (PF = 0.84–1.00, a.v.: 0.94).

#### 3.3.2. Black Tea

Another important difference in processing tea from green tea, black tea needs to be withered before rolling, then followed by fermentation and drying after rolling. Figure 2 and Supplementary Table S4 illustrated the residue variation in difenoconazole and its metabolite difenoconazole-alcohol during the processing steps of black tea. When tea leaves were sprayed and then collected at the PHI of 1, 5, 7, and 10 days, the residues of difenoconazole and its metabolite increased from 0.108–4.796 mg/kg and 0.004–0.194 mg/kg to 0.109–6.089 mg/kg and 0.005–0.383 mg/kg in withering samples with the PFs of 1.01–1.28 and 1.00–1.97, respectively. After rolling, the residues of difenoconazole were 0.127–6.707 mg/kg with the PFs of 1.04–1.18 and of difenoconazole-alcohol were 0.005–0.447 mg/kg with the PFs of 1.00–1.30. Then, difenoconazole decreased to 0.123–6.128 mg/kg and difenoconazole-alcohol increased to 0.007–0.525 mg/kg following fermentation, with the corresponding PFs of 0.91–1.02 and 1.13–1.40, respectively. After drying, the final residues of difenoconazole and difenoconazole-alcohol were at 0.417–20.711 mg/kg and 0.022–1.436 mg/kg with the PFs of 3.22–3.65 and 2.27–3.56 after drying, respectively. The PFs of difenoconazole and difenoconazole-alcohol during the total black tea processing were in the ranges of 3.86–4.76 and 3.93–7.36, respectively. Difenoconazole-alcohol increased during the black tea processing; it may be presumably converted from the parent metabolism.

**Figure 3:**
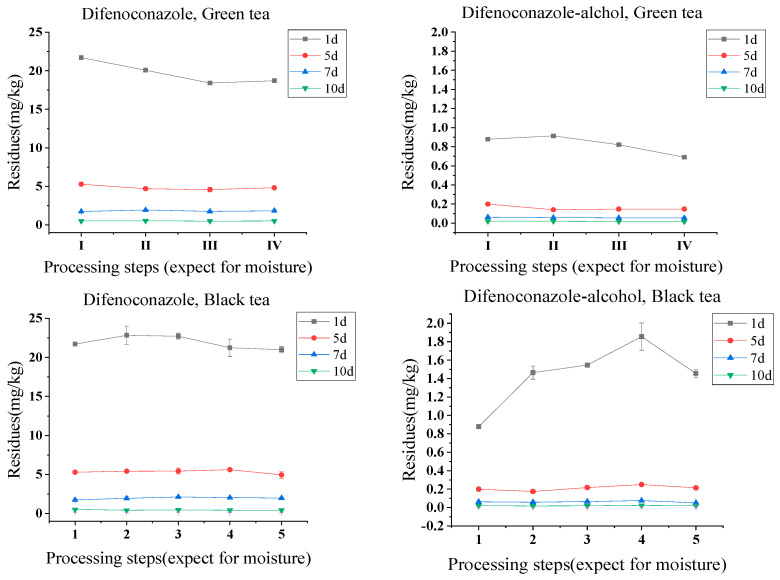
Residue variation in difenoconazole and difenoconazole-alcohol in various tea processing steps (except for moisture). Note: 1, 2, 3, 4, and 5 coordinates to the five steps of black tea processing, as fresh tea leaves, withering, rolling, fermentation, and drying, respectively; I, II, III, and IV coordinates to the four steps of green tea processing, as fresh tea leaves, fixing, rolling, and drying, respectively.

Considering the different contents of moisture in black tea processing samples (Supplementary Table S4), the average dissipation rates (DRs) for difenoconazole and difenoconazole-alcohol from fresh tea leaves to black tea were 3% and −15%, respectively. The residues of difenoconazole and difenoconazole-alcohol increased from 0.508–21.704 mg/kg and 0.020–0.878 mg/kg in the initial fresh tea leaves (PHI = 1, 5, 7, and 10 days) to 0.423–20.983 mg/kg and 0.021–1.455 mg/kg in the black tea samples, respectively. Therefore, the residues for difenoconazole and difenoconazole-alcohol shown in Figure 3 were increased during black tea processing. Overall, according to the different impacts on the different processing steps for difenoconazole and difenoconazole-alcohol, the PFs for difenoconazole were as follows: withering (PF, 0.81–1.11, a.v.: 1.00) > rolling (PF, 0.99–1.10, a.v.: 1.05) > drying (PF, 0.88–0.99, a.v.: 0.95) > fermentation (PF, 0.94–1.03, a.v.: 0.97), and in the case of difenoconazole-alcohol, were withering (PF, 0.85–1.66, a.v.: 1.08) > fermentation (PF, 1.15–1.32, a.v.: 1.22) > drying (PF, 0.70–0.85, a.v.: 0.79) = rolling (PF, 1.05–1.25, a.v.: 1.13). This indicates that the high temperature in the drying step is not the decisive factor affecting the residue changes in difenoconazole and its metabolite.

From the literature, it can be inferred that difenoconazole and its metabolite are not susceptible to degradation under high temperatures. For example, the PFs of difenoconazole in carrots from two different origins were 0.93 and 0.89 after pasteurization in Aurore, and the PFs of difenoconazole in the two carrots were 1.23 and 1.17 after sterilization at 117 °C for 7 min, respectively [29]. Meanwhile, in this experiment, the residues of difenoconazole in tea were not changed very much after a series of different processing steps, such as leaf destruction and drying at high temperatures, indicating that the residues of difenoconazole in tea were relatively stable, this may be related to the low vapor pressure of difenoconazole (3.3 × 10^−8^ Pa at 20 °C), the lower vapor pressure, the more stable is of the compound [26]. In fact, pesticides with higher vapor pressure are more prone to degradation during processing [30].

### 3.4. Leaching Rates of Difenoconazole and Difenoconazole-Alcohol from Tea

**Figure 4:**
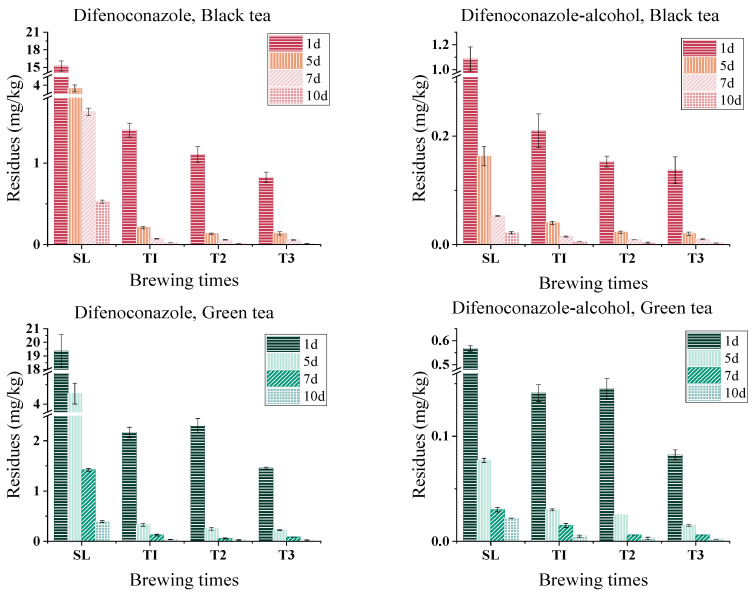
Residues of difenoconazole and difenoconazole-alcohol in different tea infusions at various brewing times (T1, T2, T3).

In most cases, tea is brewed into tea infusion for consumption, pesticide residues in tea leaves will be transmitted into tea infusion, then ingested by the body and thus affecting human health [31]. Therefore, clarifying the amount of pesticide residues in tea infusion can help identify the dietary risks. Leaching rates (LRs) of difenoconazole and difenoconazole-alcohol from tea into tea infusion were determined by the residue concentration after brewing. The results are shown in Figure 4, Supplementary Tables S5 and S6.

#### 3.4.1. Black Tea

The residues of difenoconazole in black tea infusion were much lower than those in spent black tea leaves. The residual levels in the three rounds of tea infusion varied from 0.012 mg/kg to 1.402 mg/kg. The LRs for the three rounds of brewing times were 3.8–7.6% (a.v.: 5.1%), 2.5–5.9% (a.v.: 3.7%), and 2.1–4.4% (a.v.: 3.2%), respectively, and the total LRs were 8.4–17.9% (a.v.:12%). The residues in spent tea leaves were 0.527–15.238 mg/kg with the residue rates of 82.1–91.6% (a.v.: 88%). The metabolite difenoconazole-alcohol exhibited the same trend as difenoconazole after black tea brewing, and the residue was lower in tea infusion than in spent tea leaves. The range of difenoconazole-alcohol in three black tea infusions was 0.003–0.210 mg/kg. The LRs for three brewing time rounds were 13.2–16.9% (a.v.: 15.7%), 9.5–12.1% (a.v.: 10.3%) and 8.1–11.8% (a.v.: 9.4%), and the total LR was at 31.8–38.9% (a.v.: 35.4%), respectively. Spent black tea leaves had residues ranging from 0.022 to 1.085 mg/kg, with average residual rates (RRs) of 61.1–68.2% (a.v.: 64.6%).

The residue of difenoconazole in tea infusion is much higher than that of its metabolite difenoconazole-alcohol, in contrast to the leaching rate of metabolite difenoconazole-alcohol is higher than that of difenoconazole. The fact that the spent leaves contain many more residues than the infusion dose is a characteristic shared by many pesticides [32]. And this result may be related to the water solubility of pesticides, the LRs of triazophos, endosulfan, and lambda-cyhalothrin were measured in tea infusion, where the LRs of triazophos in tea infusion was highest with its average at 29.06%, endosulfan and lambda-cyhalothrin at 5.11% and 1.73%, respectively. While, in our research, the average LR of difenoconazole in tea infusion was 15.34%, it was lower than that of triazophos, but higher than that of endosulfan and lambda-cyhalothrin, which is exactly in line with the magnitude arrangement of their respective water solubility, for example, triazophos at 39 mg/L (23 °C), difenoconazole at 15 mg/L (25 °C), and also both endosulfan and lambda-cyhalothrin lower than 5 mg/L [33].

#### 3.4.2. Green Tea

Three rounds of tea infusion produced residue levels of difenoconazole ranging from 0.025 mg/kg to 2.305 mg/kg, with the corresponding LRs of 6.5–8.6%, 4.5–9.1%, and 4.5–5.8%, and a total LR of 15.4–23.5%. In spent tea leaves, difenoconazole residue levels ranged from 0.394 to 19.345 mg/kg, with residue rates of 76.5–84.6%. The LRs of difenoconazole-alcohol during three stages of brewing were at 15.1–25.1%, 8.5–16.9%, and 4.7–10.9%, respectively, and the total residues ranged from 0.002 to 0.141 mg/kg with the total LRs at 30.4–50.6%. The residual levels in spent tea leaves ranged from 0.022 to 0.567 mg/kg, while the RRs were 49.4–69.6%.

Residues of difenoconazole in black tea infusion were lower than those in green tea infusion, while those of metabolite difenoconazole-alcohol in green tea infusion were lower. Both the black tea infusion and green tea infusion showed the same leaching trends of difenoconazole and difenoconazole-alcohol. The LR of difenoconazole-alcohol was higher than that of difenoconazole, and compared to tea infusion, the residues in spent leaves were much higher.

### 3.5. Dietary Risk Assessment of Difenoconazole and Difenoconazole-Alcohol

The original residue depositions of difenoconazole and difenoconazole-alcohol on fresh tea leaves were 16.339 and 0.212 mg/kg after spraying application, respectively. After 7 days of application, the residue of difenoconazole on fresh tea leaves at 1.562 mg/kg was higher than the MRL set for tea at 0.05 mg/kg by the EU Commission, while the residue of difenoconazole-alcohol at 0.042 mg/kg was lower than this limit. After application for 14 days, the residues of both difenoconazole and difenoconazole-alcohol were below 0.05 mg/kg, and the degradation rate was 99%.

The residues of difenoconazole and difenoconazole-alcohol in dry tea after processing into green tea were 18.711 mg/kg and 0.689 mg/kg with the degradation rates of this process at 4% and 19%, respectively. After being processed into black tea, the residues of difenoconazole and difenoconazole-alcohol in dry tea were 20.983 mg/kg and 1.455 mg/kg with degradation rates at 3% and −15%, respectively. Although the residues of difenoconazole and difenoconazole-alcohol were high in fresh tea leaves and dried tea, the leaching rates of both of them in tea infusion were low. The allowable daily intake (ADI) and the acute reference dose (ARfD) of difenoconazole were set at 0.01 mg/kg and 0.3 mg/kg, respectively, using the risk quotient method to precisely evaluate the food consumption risk. There was no ADI and ARfD data for difenoconazole-alcohol in the JMPR report, and it was not considered in the European Food Safety Authority (EFSA) revision of the MRLs set for difenoconazole in 2021. Therefore, the ADI, ARfD, and MRL for difenoconazole-alcohol in tea were calculated with reference to its parent.

Table 3 displayed the calculated RQc and RQa values of difenoconazole and difenoconazole-alcohol in black and green teas. For difenoconazole and difenoconazole-alcohol in black tea, the highest RQc values were 0.734% and 0.043%, respectively, and the highest RQa values were 0.250% and 0.038%, respectively. In green tea, the corresponding RQc values were 0.882% and 0.055%, respectively, and the RQa values were 0.030% and 0.024%, respectively. According to the findings, for difenoconazole and difenoconazole-alcohol, the RQc of green tea was higher than that of black tea, while the RQa of black tea for difenoconazole-alcohol was higher. However, the RQc and RQa for difenoconazole and difenoconazole-alcohol of both black and green teas were less than 1, suggesting that there is little chance that difenoconazole residues in tea will cause dietary intake risks to the health of consumers.

However, tea is not the only way in which humans ingest difenoconazole in their daily lives. Difenoconazole has been currently registered in China for dozens of crops, all of which are food items required for daily diets. Therefore, based on the MRL setting for difenoconazole in China and a full dietary risk assessment based on the EFSA Pesticide Residue Intake Model, the National Estimated Daily Intake (NEDI) was determined to be 0.995 mg with a risk value of 158%. The results are shown in Table 4, and it can be seen that the total dietary risk value for difenoconazole in registered crops in China is 158%, which poses some risk to human dietary health, indicating that the MRLs setting for difenoconazole in a portion of the crop may not be reasonable. The MRLs of some fruits and vegetables in China are higher than the EU Commission Regulation standard, such as strawberries, bananas, cucumbers, and shallots, which happen to be foods with a high frequency of daily human consumption, so, the MRLs setting for these fruits and vegetables may be deserving of reconsideration again.

## 4. Conclusions

The change and degradation of difenoconazole and difenoconazole-alcohol during the tea growth, processing, and brewing were investigated. The spiked recoveries of this method ranged from 71.2% to 118.6% in different tea matrices with RSDs lower than 15.9%, the LOQs for difenoconazole and difenoconazole-alcohol were 0.001 mg/kg in fresh tea leaves and dried tea, 0.0002 mg/L in tea infusion. In field trials, the half-life of difenoconazole in fresh tea leaves was 1.77 days, after 14 days of spraying, the degradation rate of difenoconazole and difenoconazole-alcohol reached 99%. In the process of processing both black and green tea, the major impact processing step in green tea on the residues of difenoconazole and its metabolite difenoconazole-alcohol is the rolling, while in black tea, rolling is the major impact processing step on difenoconazole, and fermentation is the most effective impact on difenoconazole-alcohol. Ultimately, after the total processing process, difenoconazole and difenoconazole-alcohol did not change much in both black and green tea, indicating that difenoconazole is a relatively stable pesticide. In contrast, the range of difenoconazole leaching rates in green tea infusion was 15.4–23.5%, while the range for black tea infusion was 8.4–17.9%, respectively. The leaching rates of the metabolite difenoconazole-alcohol in infusions of green and black tea ranged from 30.4 to 50.6% and 31.8 to 38.9%, which were higher than those of difenoconazole. According to the dietary risk assessment, the RQc and RQa of difenoconazole and difenoconazole-alcohol were far below 1%, indicating that there was no potential health harm to humans. However, the total dietary risk assessment of difenoconazole was alarming the MRL, so, the settings of MRL for difenoconazole in the registered and other unregistered crops need to be further modified and refined.

## Figures and Tables

**Table 2:** The spiked average recoveries (A.R), standard deviation (SDs), relative standard deviation (RSDs), and limits of quantification (LOQs) for difenoconazole and difenoconazole-alcohol in different tea matrices.

Matrix	Spiked Level (mg/kg)	Difenoconazole			Difenoconazole-Alcohol		
		A.R ± SDs %, n = 6	RSDs %	LOQs (mg/kg)	A.R ± SDs %, n = 6	RSDs %	LOQs (mg/kg)
Fresh tea leaves	1	101.9 ± 4.5	4.4	0.001	104.3 ± 4.9	4.7	0.001
	0.1	102.3 ± 12.0	11.7		93.4 ± 8.5	9.1	
	0.01	92.6 ± 6.0	6.5		90.5 ± 9.4	10.4	
	0.001	92.1 ± 12.2	13.2		84.4 ± 7.3	8.6	
Green tea	1	81.0 ± 11.5	14.2	0.001	75.8 ± 12.0	15.9	0.001
	0.1	92.4 ± 7.1	7.7		83.7 ± 4.8	5.7	
	0.01	73.7 ± 6.5	8.8		71.2 ± 7.2	10.1	
	0.001	81.4 ± 5.0	6.2		84.3 ± 4.3	5.1	
Black tea	1	98.3 ± 5.9	6.0	0.001	114.9 ± 17.9	15.6	0.001
	0.1	96.7 ± 5.1	5.3		81.1 ± 8.8	10.9	
	0.01	77.6 ± 3.1	3.9		80.6 ± 5.5	6.8	
	0.001	118.6 ± 8.0	6.7		82.8 ± 6.3	7.1	
Green tea infusion (mg/L)	0.2	87.4 ± 3.6	4.1	0.0002	100.5 ± 8.0	7.9	0.0002
	0.02	83.3 ± 4.3	5.2		95.0 ± 4.1	4.3	
	0.002	96.5 ± 8.1	8.4		89.8 ± 4.5	5.1	
	0.0002	73.4 ± 8.9	12.2		88.5 ± 12.7	14.4	
Black tea infusion (mg/L)	0.2	101.1 ± 8.7	8.6	0.0002	82.3 ± 12.1	14.7	0.0002
	0.02	80.7 ± 6.7	8.3		81.2 ± 8.0	9.9	
	0.002	91.3 ± 10.1	11.0		97.5 ± 7.4	7.6	
	0.0002	88.3 ± 3.0	3.4		87.3 ± 2.5	2.8	

**Table 3:** The calculated RQc and RQa for difenoconazole and difenoconazole-alcohol in black and green teas.

Compound	Item	Difenoconazole	Difenoconazole-Alcohol
Black tea	FI (kg)	0.013	0.013
	STMR (mg/kg)	2.049	0.055
	LR (%)	17.9	38.9
	bw (kg)	65	65
	ADI (mg/kg b.w.)	0.010	0.010
	RQc (%)	0.734	0.043
	HR (mg/kg)	20.983	1.455
	ARfD (mg/kg b.w.)	0.300	0.300
	RQa (%)	0.250	0.038
Green tea	FI (kg)	0.013	0.013
	STMR (mg/kg)	1.876	0.054
	LR (%)	23.5	50.6
	bw (kg)	65	65
	ADI (mg/kg b.w.)	0.010	0.010
	RQc (%)	0.882	0.055
	HR (mg/kg)	19.196	0.698
	ARfD (mg/kg b.w.)	0.300	0.300
	RQa (%)	0.300	0.024

Notes: FI (kg) is the food intake amount; LR is the leaching rate; STMR is the median residue and HR is the highest residue in supervised trials; bw is the global average body weight (65 kg); ADI and ARfD are the acceptable daily intake and acute reference dose; RQa and RQc indicate the acute risks and chronic risks.

**Table 4:** The risks for total dietary intake of difenoconazole.

Food Category	Food Intake /kg	Reference MRLs mg/kg	Commodity	NEDI /mg	Acceptable Daily Intake /mg	Risk Probability /%
Rice and its products	0.240	0.5	Paddy	0.120	ADI×63	-
Wheat flour and its products	0.139	0.1	Wheat	0.014		
Dark color vegetables	0.092	15	Celery	1.373		
Light color vegetables	0.184	2	Scallion	0.367		
Fruit	0.046	5	Strawberry	0.229		
Vegetable oil	0.033	2	Sesame	0.065		
Drinks	0.013	10	Tea	0.130		
SUM	-	-	-	0.995	0.63	158

## Data Availability

The original contributions presented in the study are included in the article and Supplementary Material, further inquiries can be directed to the corresponding authors.

## Supplementary Materials

The following supporting information can be downloaded at: https://www.mdpi.com/article/10.3390/foods13071123/s1, Supplementary Figure S1: Chemical structures of difenoconazole and its metabolite difenoconazole-alcohol. Figure S2: The UPLC-MS/MS Chromatograms of difenoconazole and difenoconazole-alcohol in solvent standard of 0.05 mg/L, real fresh tea leaf, black tea, and tea infusion samples. Table S1: Gradient program for chromatographic elution and tandem mass spectrum conditions for difenoconazole and difenoconazole-alcohol by UPLC-MS/MS. Table S2: The average residues, dissipation rates, dissipation equations, R_2_ and t_1/2_ of difenoconazole and difenoconazole-alcohol in fresh tea leaves. Table S3: The residues and processing factors of difenoconazole and difenoconazole-alcohol in green tea processing. Table S4: The residues and processing factors of difenoconazole and difenoconazole-alcohol in black tea processing. Table S5: Residues and leaching rates of difenoconazole and difenoconazole-alcohol during black tea brewing. Table S6: Residues and leaching rates of difenoconazole and difenoconazole-alcohol during green tea brewing.
