# Supplementary material for: Residue Degradation and Risk Assessment of Difenoconazole and Its Metabolite during Tea Growing, Processing and Brewing by Ultra-Performance Liquid Chromatography–Tandem Mass Spectrometry Determination
Source: Foods. 2024 Apr 7;13(7):1123. doi: 10.3390/foods13071123 (PMC11011539; doi:10.3390/foods13071123)
Supplement: Supplementary file 1 [file foods-13-01123-s001.zip › foods-2873013-supplementary.pdf]

Supporting Information

Residue Degradation and Risk Assessment of Difenoconazole and Its Metabolite During Tea Growing, Processing and Brewing by Ultra Performance Liquid Chromatography–Tandem Mass Spectrometry Determination

Min Wang [1,2,3,†], Yating Ning [1,4,†], Yue Hu [1,4], Xinyi Cui [2], Fengjian Luo [1,5], Li Zhou [1,5], Miao Yu [3,*] and Xinzhong Zhang [1,5,*]

[1] Research Center of Quality Safety for Agricultural Products, Tea Research Institute, Chinese Academy of Agricultural Sciences, Hangzhou 310008, China
[2] College of Horticulture and Landscape, Tianjin Agricultural University, Tianjin 300384, China
[3] Zhejiang Provincial Plant Protection Quarantine and Pesticide Management Institute, Hangzhou 310000, China
[4] Graduate School of Chinese Academy of Agricultural Sciences, Beijing 100081, China
[5] Key Laboratory of Tea Quality and Safety Control, Ministry of Agriculture and Rural Affairs, Hangzhou 310008, China

*Correspondence: zhangxinzhong@tricaas.com; Tel.: +86-571-87963516; ORCID ID: 0000-0001-7369-5761; Fax: +86-571-86650331; yumiao2020@sina.com. Tel: +86-571-86757007.

[†] Co-first author. Min Wang and Yating Ning are equal contribution to this paper.

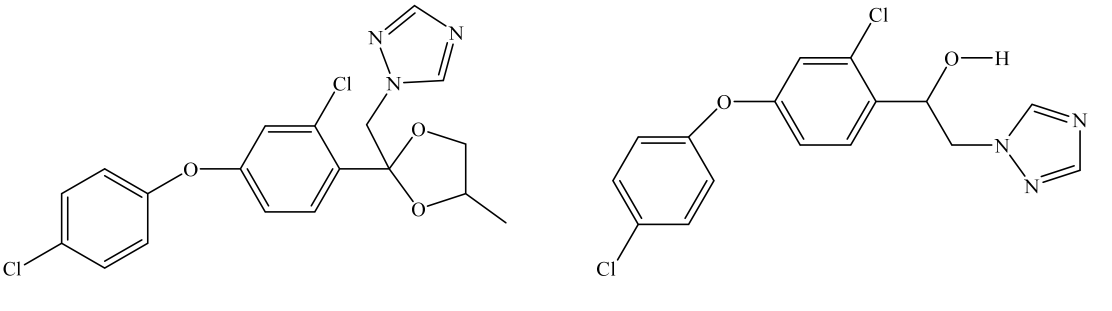

Suppl Figure S1. Chemical structures of difenoconazole and its metabolite difenoconazole-alcohol.

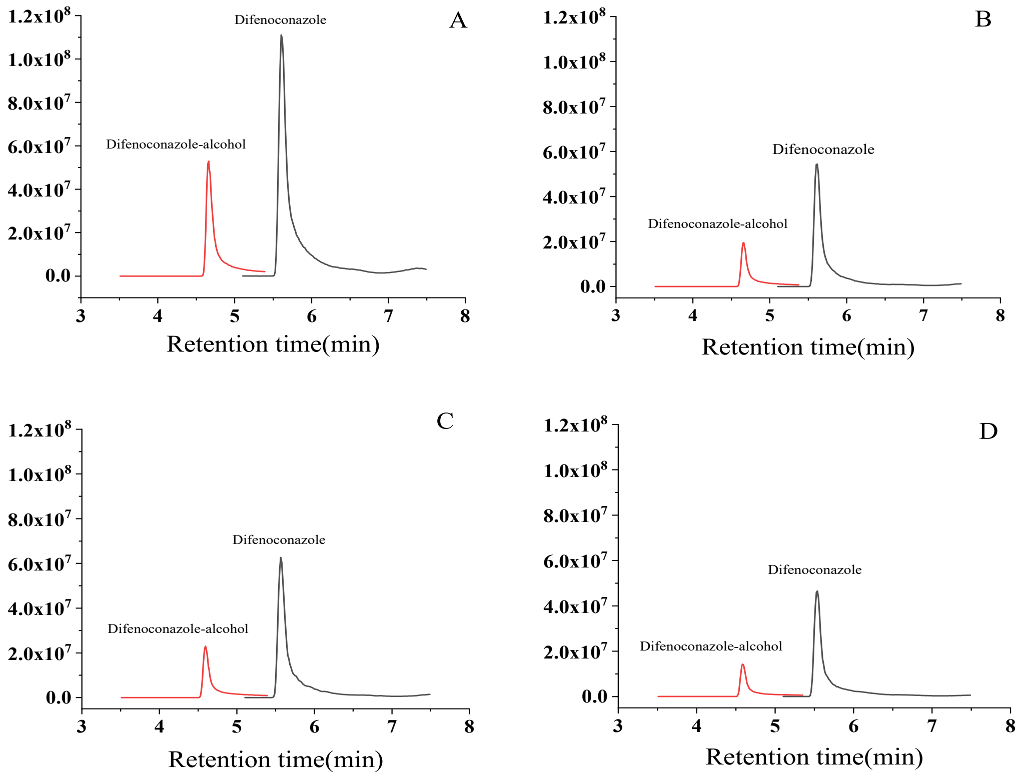

Suppl Figure S2. The UPLC-MS/MS Chromatograms of difenoconazole and difenoconazole-alcohol in solvent standard of 0.05 mg/L (A), real fresh tea leaf (B), black tea (C) and tea infusion (D) samples.

Suppl Table S1. Gradient program for chromatographic elution and tandem mass spectrum conditions for difenoconazole and difenoconazole-alcohol by UPLC-MS/MS.

Time /min	A %	B %	Compound	Rt /min	Precursor ion *m/z*	CV[a] /V	MRM *m/z* > *m/z*	CE[b] /eV
0	10	90	difenoconazole	5.65	406	35	406 > 337[c]	15
1.5	70	30					406 > 251 [d]	30
5.0	85	15	difenoconazole-alcohol	4.72	350	35	350 > 70 [c]	20
7.5	99	1					350 > 280.5 [d]	15
9.0	100	0						
9.8	100	0						
10.3	10	90	Note: [a]. CV means cone voltage; [b]. CE means collision energy; [c]. for quantification; [d]. for conformation					
12.0	10	90						

Suppl Table S2. The average residues, dissipation rates, dissipation equations, R^2 and $t_{1/2}$ of difenoconazole and difenoconazole-alcohol in fresh tea leaves.

Compound	difenoconazole		difenoconazole-alcohol	
Time (d)	Residues (mg/kg, ±SD)	Dissipation Rate (%)	Residues (mg/kg, ±SD)	Dissipation Rate (%)
0 (2 h)	16.339±1.418	/	0.212±0.028	/
1	15.759±0.256	3.5	0.705±0.020	-232.4
2	8.753±0.304	46.4	0.542±0.029	-155.7
3	7.883±0.266	51.8	0.435±0.017	-105.2
5	2.886±0.331	82.3	0.079±0.011	62.9
7	1.562±0.169	90.4	0.042±0.002	80.1
10	0.348±0.032	97.9	0.005±0.003	97.9
14	0.018±0.003	99.9	0.001±0.001	99.5
Dissipation equation	$C = 17.771e^{-0.392t}$		-	
R^2	0.9641		-	
$t_{1/2}$ (d)	1.77		-	

Suppl Table S3. The residues and processing factors of difenoconazole and difenoconazole-alcohol in green tea processing

Processing steps	Time (d)	Moisture content (%)	difenoconazole<br>Residues (mg/kg, ± SD) (PF)	difenoconazole (expect for moisture)<br>Residues (mg/kg, ± SD) (PF)	difenoconazole-alcohol<br>Residues (mg/kg, ±SD) (PF)	difenoconazole-alcohol (expect for moisture)<br>Residues (mg/kg, ± SD) (PF)
Fresh tea leaves	1	77.9	4.796±0.025 (-)	21.704±0.113 (-)	0.194±0.002 (-)	0.878±0.009 (-)
	5	80.6	1.026±0.027 (-)	5.286±0.138 (-)	0.038±0.000 (-)	0.199±0.002 (-)
	7	76.6	0.408±0.002 (-)	1.743±0.003 (-)	0.015±0.000 (-)	0.062±0.001 (-)
	10	78.8	0.108±0.000 (-)	0.508±0.006 (-)	0.004±0.000 (-)	0.020±0.000 (-)
Fixing	1	64.2	7.186±0.189 (1.50)	20.072±0.528 (0.92)	0.327±0.011 (1.69)	0.913±0.030 (1.04)
	5	66.6	1.571±0.074 (1.53)	4.703±0.220 (0.89)	0.047±0.002 (1.24)	0.140±0.006 (0.70)
	7	63.2	0.712±0.023 (1.75)	1.934±0.064 (1.11)	0.021±0.001 (1.40)	0.058±0.002 (0.94)
	10	66.1	0.180±0.003 (1.67)	0.530±0.009 (1.04)	0.007±0.000 (1.75)	0.020±0.000 (1.00)
Rolling	1	65	6.441±0.278 (0.90)	18.409±0.783 (0.92)	0.287±0.024 (0.88)	0.821±0.069 (0.90)
	5	64.8	1.613±0.103 (1.03)	4.584±0.294 (0.97)	0.052±0.003 (1.11)	0.147±0.010 (1.05)
	7	62.7	0.656±0.012 (0.92)	1.757±0.031 (0.91)	0.021±0.000 (1.00)	0.055±0.001 (0.95)
	10	63.7	0.181±0.002 (1.01)	0.498±0.005 (0.94)	0.007±0.000 (1.00)	0.018±0.001 (0.90)
Drying	1	5	17.776±1.336 (2.76)	18.711±1.406 (1.02)	0.655±0.047 (2.28)	0.689±0.049 (0.84)
	5	5.75	4.542±0.151 (2.82)	4.819±0.160 (1.05)	0.138±0.005 (2.65)	0.147±0.005 (1.00)
	7	5.5	1.731±0.140 (2.64)	1.832±0.149 (1.04)	0.051±0.007 (2.43)	0.054±0.008 (0.98)
	10	6.25	0.483±0.000 (2.67)	0.515±0.011 (1.03)	0.016±0.000 (2.29)	0.017±0.000 (0.94)
Total PF[b] (Degradation rate, %)	1	-	3.71 (-271)	0.86 (14)	3.38 (-238)	0.78 (22)
	5	-	4.43 (-343)	0.91 (9)	3.63 (-263)	0.74 (26)
	7	-	4.24 (-324)	1.05 (-5)	3.40 (-240)	0.87 (13)
	10	-	4.47 (-347)	1.01 (-1)	4.00 (-300)	0.85 (15)
	Average	-	4.21 (-321)	0.90 (4)	3.60 (-260)	0.81 (19)

Suppl Table S4. The residues and processing factors of difenoconazole and difenoconazole-alcohol in black tea processing

Processing steps	Time (d)	Moisture content (%)	difenoconazole Residues (mg/kg, ± SD) (PF)	difenoconazole (expect for moisture) Residues (mg/kg, ± SD) (PF)	difenoconazole-alcohol Residues (mg/kg, ± SD) (PF)	difenoconazole-alcohol (expect for moisture) Residues (mg/kg, ± SD) (PF)
Fresh tea leaves	1	77.9	4.796±0.025 (-)	21.704±0.113 (-)	0.194±0.002 (-)	0.878±0.009 (-)
	5	80.6	1.026±0.027 (-)	5.286±0.138 (-)	0.038±0.000 (-)	0.199±0.002 (-)
	7	76.6	0.408±0.002 (-)	1.743±0.003 (-)	0.015±0.000 (-)	0.062±0.001 (-)
	10	78.8	0.108±0.000 (-)	0.508±0.006 (-)	0.004±0.000 (-)	0.020±0.000 (-)
Withering	1	73.0	6.089±0.324 (1.27)	23.240±1.238 (1.07)	0.383±0.018 (1.97)	1.461±0.070 (1.66)
	5	73.3	1.248±0.016 (1.22)	5.403±0.071 (1.02)	0.040±0.001 (1.05)	0.175±0.004 (0.88)
	7	73.0	0.521±0.057 (1.28)	1.929±0.210 (1.11)	0.015±0.002 (1.00)	0.057±0.006 (0.92)
	10	76.9	0.109±0.007 (1.01)	0.409±0.027 (0.81)	0.005±0.000 (1.25)	0.017±0.001 (0.85)
Rolling	1	71.7	6.707±0.115 (1.10)	23.050±0.394 (0.99)	0.447±0.005 (1.17)	1.537±0.016 (1.05)
	5	71.4	1.303±0.095 (1.04)	5.429±0.396 (1.00)	0.052±0.002 (1.30)	0.218±0.008 (1.25)
	7	71.1	0.613±0.026 (1.18)	2.121±0.088 (1.10)	0.019±0.001 (1.27)	0.062±0.005 (1.09)
	10	76.0	0.127±0.009 (1.17)	0.446±0.033 (1.09)	0.005±0.000 (1.00)	0.019±0.001 (1.12)
Fermentation	1	71.6	6.128±0.319 (0.91)	21.655±1.126 (0.94)	0.525±0.042 (1.17)	1.857±0.148 (1.21)
	5	71.2	1.333±0.021 (1.02)	5.603±0.088 (1.03)	0.059±0.000 (1.13)	0.250±0.002 (1.15)
	7	70.2	0.604±0.017 (0.99)	2.025±0.057 (0.95)	0.022±0.000 (1.16)	0.073±0.001 (1.18)
	10	76.2	0.123±0.016 (0.96)	0.426±0.057 (0.96)	0.007±0.000 (1.40)	0.025±0.001 (1.32)
Drying	1	1.5	20.711±0.409 (3.38)	20.983±0.414 (0.97)	1.436±0.041 (2.74)	1.455±0.042 (0.78)
	5	1.4	4.862±0.412 (3.65)	4.941±0.419 (0.88)	0.210±0.012 (3.56)	0.213±0.012 (0.85)
	7	1.3	1.944±0.104 (3.22)	1.969±0.105 (0.97)	0.050±0.004 (2.27)	0.051±0.004 (0.70)
	10	1.6	0.417±0.012 (3.39)	0.423±0.012 (0.99)	0.022±0.001 (3.14)	0.021±0.001 (0.84)
Total PF[a] (Degradation rate, %)	1	-	4.32 (-332)	0.97 (3)	7.36 (-640)	1.66(-66)
	5	-	4.74 (-374)	0.93 (7)	5.53 (-453)	1.07 (-7)
	7	-	4.76 (-376)	1.13 (-13)	3.93 (-233)	0.82 (18)
	10	-	3.86 (-286)	0.83 (17)	5.25 (-450)	1.05 (-5)
	Average	-	4.42 (-342)	0.98 (3)	5.44 (-444)	1.16 (-15)

Suppl Table S5. Residues and leaching rates of difenoconazole and difenoconazole-alcohol during black tea brewing.

Compound	Time (d)	First infusion (T1) Residues (mg/kg, ±SD)	First infusion (T1) LR_1 (%)	Second infusion (T2) Residues (mg/kg, ±SD)	Second infusion (T2) LR_2 (%)	Third infusion (T3) Residues (mg/kg, ±SD)	Third infusion (T3) LR_3 (%)	LRt (%)	Spent leaves (SL) Residues (mg/kg, ±SD)	Spent leaves (SL) RR (%)
difenoconazole	1	1.402±0.086	7.6	1.104±0.096	5.9	0.823±0.064	4.4	17.9	15.238±0.877	82.1
	5	0.210±0.016	4.9	0.135±0.008	3.1	0.138±0.020	3.2	11.3	3.815±0.198	88.7
	7	0.073±0.004	4.0	0.059±0.004	3.3	0.058±0.005	3.2	10.5	1.627±0.046	89.5
	10	0.022±0.001	3.8	0.014±0.001	2.5	0.012±0.001	2.1	8.4	0.527±0.018	91.6
difenoconazole-alcohol	1	0.210±0.031	13.2	0.153±0.010	9.6	0.137±0.025	9.0	31.8	1.085±0.096	68.2
	5	0.040±0.003	16.2	0.023±0.002	9.5	0.020±0.003	8.1	33.8	0.163±0.018	66.2
	7	0.015±0.001	16.9	0.009±0.000	10.1	0.010±0.001	11.8	38.9	0.053±0.001	61.1
	10	0.006±0.000	16.4	0.004±0.001	12.1	0.003±0.000	8.7	37.2	0.022±0.002	62.8

Suppl Table S6. Residues and leaching rates of difenoconazole and difenoconazole-alcohol during green tea brewing.

Compound	Time (d)	First infusion (T1) Residues (mg/kg, ±SD)	First infusion (T1) LR_1 (%)	Second infusion (T2) Residues (mg/kg, ±SD)	Second infusion (T2) LR_2 (%)	Third infusion (T3) Residues (mg/kg, ±SD)	Third infusion (T3) LR_3 (%)	LRt (%)	Spent leaves (SL) Residues (mg/kg, ±SD)	Spent leaves (SL) RR (%)
difenoconazole	1	2.165±0.104	8.6	2.305±0.138	9.1	1.459±0.019	5.8	23.5	19.345±1.213	76.5
	5	0.329±0.028	6.5	0.249±0.030	4.5	0.226±0.008	4.5	15.4	4.272±0.267	84.6
	7	0.126±0.018	7.4	0.058±0.009	5.0	0.085±0.001	4.9	17.3	1.422±0.028	82.7
	10	0.035±0.003	7.2	0.028±0.004	5.9	0.025±0.005	5.2	18.4	0.394±0.017	81.6
difenoconazole-alcohol	1	0.141±0.008	15.1	0.145±0.010	15.5	0.082±0.005	8.8	39.4	0.567±0.012	60.6
	5	0.030±0.001	20.2	0.025±0.001	16.9	0.015±0.001	10.5	47.6	0.077±0.002	52.4
	7	0.015±0.002	25.1	0.006±0.000	14.5	0.006±0.000	10.9	50.6	0.030±0.002	49.4
	10	0.005±0.001	17.3	0.003±0.001	8.5	0.002±0.000	4.7	30.4	0.022±0.000	69.6
